# Impact of circulating tumor DNA in hepatocellular and pancreatic carcinomas

**DOI:** 10.1007/s00432-020-03219-5

**Published:** 2020-04-27

**Authors:** Sameer A. Dhayat, Zixuan Yang

**Affiliations:** Department of General, Visceral and Transplantation Surgery, University Hospital Muenster, Albert-Schweitzer-Campus 1 (W1), 48149 Munster, Germany

**Keywords:** Hepatocellular carcinoma, Pancreatic cancer, Circulating tumor DNA, Next-generation sequencing, Digital droplet PCR

## Abstract

Hepatocellular carcinoma (HCC) and pancreatic cancer (PC) belong to the most lethal malignancies worldwide. Despite advances in surgical techniques and perioperative multidisciplinary management, the prognosis of both carcinoma entities remains poor mainly because of rapid tumor progression and early dissemination with diagnosis in advanced tumor stages with poor sensitivity to current therapy regimens. Both highly heterogeneous visceral carcinomas exhibit unique somatic alterations, but share common driver genes and mutations as well. Recently, circulating tumor DNA (ctDNA) could be identified as a liquid biopsy tool with huge potential as non-invasive biomarker in early diagnosis and prognosis. CtDNA released from necrotic or apoptotic cells of primary tumors, metastasis, and circulating tumor cells can reveal genetic and epigenetic alterations with tumor-specific and individual mutation and methylation profiles. In this article, we focus on clinical impact of ctDNA as potential biomarker in patients with HCC and PC.

## Background

Hepatocellular carcinoma (HCC) and pancreatic cancer (PC) represent two of the most challenging visceral malignancies in oncology with rising incidence and lack of reliable biomarkers for early diagnosis, prognosis, and therapy response. PC and HCC are estimated to become the second and third respective leading causes of cancer-related death in western countries by 2030 (Rahib et al. 2014; Siegel et al. 2019). As both carcinoma entities share some common risk factors, either environmental or genetic, combined analyses may provide useful information. Hepatocellular carcinogenesis is a multistep process occurring in one-third of patients with liver cirrhosis, on the background of chronic infection with hepatitis B or C virus (HBV, HCV), alcoholic or non-alcoholic steatohepatitis, and obesity (Villanueva 2019). PC occurs with increased frequency among individuals with tobacco smoking, type 2 diabetes, obesity, chronic pancreatitis or hereditary risk factors (Ryan et al. 2014). In both carcinoma entities, the majority of patients are diagnosed at advanced stages with a low 5-year survival rate of less than 10–20% and a high 5-year recurrence rate of 70–80%, even following oncological tumor resection (Siegel et al. 2019). Therefore, early diagnosis at surgically manageable stages and early recurrence detection would have a tremendous impact on survival of patients with HCC or PC. However, current screening of proteomic serum markers, such as alpha-fetoprotein (AFP) in HCC or carcinoembryonic antigen (CEA) and carbohydrate antigen (CA) 19.9 in PC have not shown to be effective due to their reduced predictive values (Bolondi et al. 2013; Gamil et al. 2018; Poruk et al. 2013). In addition, imaging techniques failed to detect early lesions or to distinguish between benign and malignant lesions, so far. In addition to the risk of neoplastic needle tract seeding, minimal invasive solid biopsies by endoscopic-ultrasound-guided fine needle aspiration in PC or percutaneous needle biopsy in HCC cannot accurately track dynamic changes due to high tumor heterogeneity (Stigliano and Burroughs 2005; Yoshida et al. 2019). This molecular heterogeneity is the reason for the large variation in individual patient`s prognosis and response to chemotherapy.

Cytotoxic chemotherapy agents continue to form the backbone for the treatment of advanced PC limited to the pyrimidine antimetabolites gemcitabine and 5-fluorouracil (5-FU), Topoisomerase I inhibition by irinotecan, the DNA crosslinking agents oxaliplatin and cisplatin, and the tubulin inhibitor paclitaxel (Burris et al. 1997; Reni et al. 2005; Stathopoulos et al. 2006). However, the median survival remains 6–11 months. Since 2007, gemcitabine-based combination chemotherapies with the selective epidermal growth factor receptor (EGFR) tyrosine kinase inhibitor erlotinib could improve the overall survival in locally advanced, unresectable, or metastatic PC (Moore et al. 2007). 5-FU-based FOLFIRINOX therapy including calcium folinate, irinotecan, and oxaliplatin since 2011 as well as the addition of nanoparticle albumin-bound paclitaxel to gemcitabine since 2013 significantly prolonged the overall survival and progression-free survival compared with mono-gemcitabine therapy in locally advanced and metastatic PC (Conroy et al. 2011; Von Hoff et al. 2013). Due to its greater toxicity, FOLFIRINOX was recommended for PC patients in good physical condition. High resistance to current chemotherapy regimens still affects the treatment of PC and HCC (Chin et al. 2018). However, better therapy response with extended survival time of more than 2 years in small subgroups of patients with advanced PC seems to be connected with exceptionally favorable prognostic factors and molecular characteristics (Collisson et al. 2011; Cui et al. 2012).

Despite the identification of many frequently mutated genes as potential therapeutic targets in HCC, the multikinase inhibitor Sorafenib that inhibits vascular endothelial growth factor receptor (VEGFR) 1–3, platelet-derived growth factor receptor (PDGFR) beta, c-KIT, and RAF/mitogen-activated protein/MEK was the sole drug approved for the treatment of advanced HCC between 2007 and 2016 with a response rate of less than 5% and an extended median overall survival of 2.5 months (Llovet et al. 2008). Currently, two first-line alternatives to Sorafenib are approved. The first Lenvatinib is a multikinase inhibitor as well, that inhibits VEGFR 1–3, PDGFR alpha, fibroblast growth factor receptor 1–4, c-Kit proto-oncogene receptor tyrosine kinase, and RET proto-oncogene with an improved median overall survival of 13.6 months versus 12.3 months. The other one is the monoclonal antibody Nivolumab, that inhibits the immune checkpoint molecule programmed cell death protein 1 and showed a response rate of 23% in Sorafenib naïve HCC patients (Forner et al. 2018). Therefore, the most effective treatment of heterogeneous cancers like HCC and PC might be a tailored combination of drugs targeting specific genomic and epigenomic alterations. Routine molecular testing is still performed in clinical diagnostic for targeted therapy and prognostic stratification in cancer entities, such as breast cancer, melanoma and leukemia (El-Deiry et al. 2019). In patients with diagnosed colorectal carcinoma, the decision for an anti-EGFR antibody therapy is based on routine mutation analysis of kirsten rat sarcoma viral oncogene homolog (KRAS), neuroblastoma RAS viral oncogene homolog, and B-Raf proto-oncogene serine/threonine kinase gene resulting in a valine-to-glutamate change at the residue 600 (Karapetis et al. 2008). In addition to environmental factors, recent genome-wide association studies revealed that genetic and epigenetic abnormalities might be significant determinants of HCC or PC susceptibility with important influence on the individual predisposition to disease progression, e.g. chronic inflammation, and resulting carcinogenesis. Moreover, several recent studies have shown that identification of molecular biomarkers and real-time monitoring of disease and therapy efficacy in PC and HCC could be achieved by liquid biopsies (Tables 1 and 2). In this review, we discuss recent studies focusing on detection and clinical impact of circulating DNA mutation and methylation as potential biomarker for early diagnosis, prognosis, and therapy response in HCC and PC.

**Table 1:** Circulating Tumor DNA in Hepatocellular Carcinoma

References	Origin	HCC (*n*)	Technique	Circulating markers	ctDNA mutation (%)	tiDNA mutation (%)		Mutation in liquid and tumor tissue			Carcinoma vs. healthy		Biomarker
								Sensitivity (%)	Specificity (%)	Concordance ctDNA/tiDNA (%)	Sensitivity (%)	Specificity (%)	
Huang et al. (2016)	Plasma	48	ddPCR Sanger seq	TERT CTNNB1 TP53	24.4 12.2 14.6	19.5 0 4.9		62.5 NA 50.0	84.9 87.8 87.2	80.5 87.8 85.4	NA	NA	NA
Liao et al. (2016)	Plasma	41	NGS	TERT CTNNB1 TP53	4.9 9.8 4.9	70.7 26.8 65.9		6.9 27.3 2.3	95.1 96.7 100.0	34.1 78.0 39.0	NA	NA	Poor RFS
An et al. (2019)	Plasma	26	NGS	354 genes (TP53)	96.2 50	NA 30.8		NA	NA	88.5 69.2	NA	NA	DFS (HR = 7.66; *p* < 0.001)
Szymanska et al. (2004)	Plasma	17	PCR–RFLP SOMA	TP53	41	34.5		71.4	61.5	76.7	NA	NA	NA
Lin et al. (2011)	Urine	17	LNA PCR	TP53	52.9	42.9		66.7	0.0	28.6	53.0	75.0	NA
Kimbi et al. (2005)	Serum	158	PCR and Seq	TP53	17.7	NA		NA	NA	NA	18	83.3	NA
Marchio et al. (2018)	Plasma	149	ddPCR	TP53	24.8	NA		NA	NA	NA	NA	NA	NA
Ikeda et al. (2018b)	Plasma	14	NGS	68 genes (TP53)	79 57	NA		NA	NA	NA	NA	NA	NA
Ikeda et al. (2018a)	Blood	26	NGS	70 genes (TP53)	88.5 61.5	NA		NA	NA	NA	NA	NA	NA
Riviere et al. (2018)	Plasma	31	NGS	68 genes (TP53)	74 61.2	NA		NA	NA	NA	NA	NA	NA
He et al. (2019)	Plasma	29	NGS	35 genes (TP53)	96.4 50	NA		75 NA	NA	75 33	NA	NA	NA
Kaseb et al. (2019)	Plasma	219	NGS	70 genes	87.8	NA		NA	NA	NA	NA	NA	NA
Xiong et al. (2019)	Plasma	37	NGS	TP53	64	NA		NA	NA	NA	65	100	NA
Xu et al. (2017)	Plasma	1098	MSP	target panel	NA	NA		NA	NA	NA	85.7	94.3	OS (HR = 2.41; *p* < 0.001)
Iyer et al. (2010)	Plasma	28	MSP	mp15 mp16 mAPC mFHIT	10.7 46.4 53.5 67.8	14.2 71.4 64.2 75		50 60 77.8 94.7	95.8 87.5 90 66.7	89.2 67.9 82.1 85.7	NA	NA	NA
Wong et al. (1999)	Plasma	22	MSP	mp16	59.1	72.7		NA	NA	81	NA	NA	NA
Wong et al. (2000)	Plasma	25	MSP	mp15 / mp16	87	92		NA	NA	74	NA	NA	NA
Wong et al. (2003)	Plasma	29	MSP	mp16INK4a	79.3	66.7		NA	NA	46.7	NA	NA	NA
Huang et al. (2014)	Serum	66	MSP	mINK4A	65.3	NA		NA	NA	NA	65.3	87.2	NA
Huang et al. (2011)	Plasma	72	MSP	mAPC, mGSTP1, mRASSF1A, and mSFRP1	84.7	NA		NA	NA	NA	92.7	81.9	OS (HR = 3.26; *p* = 0.003)
Chan et al. (2008)	Serum	63	MSP	mRASSF1A	93	NA		NA	NA	NA	77	89	poor DFS
Yeo et al. (2005)	Plasma	40	MSP	mRASSF1A	42.5	92.5		45.9	100.0	50.0	NA	NA	NA
Hu et al. (2010)	Serum	35	MSP	mRASSF1A	40	88.6		45.2	100	51.4	70–100	52–100	NA
Mohamed et al. (2012)	Serum	40	MSP	mRASSF1A	90	NA		NA	NA	NA	75	80	NA
Mansour et al. (2017)	Serum	45	MSP	mRASSF1A	87.7	NA		NA	NA	NA	86.7	72.5	correlation with tumor size (*r* = 0.728; *p* < 0.001)
Liu et al. (2017)	Serum	105	MSP	mRASSF1A hypomLINE-1	73.3 66.7	NA		NA	NA	NA	NA	NA	poor DFS and OS
Tangkijvanich et al. (2007)	Serum	85	MSP	hypomLINE-1	70.4	NA		NA	NA	NA	NA	NA	increased HCC risk (OR = 1.74), poor OS
Oussalah et al. (2018)	Plasma	51	MSP	mSEPT9	83.0	NA		NA	NA	NA	94.1	84.4	NA
Sun et al. (2013)	Serum	43	MSP	mTFPI2	46.5	NA		NA	NA	NA	80.8	80	NA
Wu et al. (2017)	Plasma	237	MSP	mTBX2	75.5	NA		NA	NA	NA	75.5	41.2	Increased HCC risk (OR = 2.39)
Iizuka et al. (2011)	Serum	108	MSP	mBASP1 mCCND2 mAPC mCFTR mRASSF1A	NA	NA		NA	NA	NA	62.0 64.8 17.6 56.5 83.3	78.6 42.9 78.6 83.9 58.9	NA
Zhang et al. (2013)	Serum	31	MSP	mDBX2 mTHY1	NA	NA		NA	NA	NA	88.9 85.2	87.1 80.7	NA
Ji et al. (2014)	Serum	121	MSP	mMT1M mMT1G mMT1M/G	48.8 70.2	NA		NA	NA	NA	48.8 70.2 90.9	93.5 87.1 83.9	Correlation with tumor size (*r* = 0.321; *p* < 0.001)
Han et al. (2014)	Serum	160	MSP	mTGR5 mTGR5 and mAFP	48.1 NA	NA		NA	NA	NA	NA 65.0	NA 85.2	NA
Wang et al. (2006)	Serum	32	MSP	GSTP1	50	88.5		60.9	100	65.4	50.0	62.5	NA
Wen et al. (2015)	Plasma	36	MSP	mRGS10, mST8SIA6, mVIM, and mRUNX2	85.6	NA		NA	NA	NA	94.0	89.0	NA

ctDNA: circulating tumor DNA, ddPCR: droplet digital polymerase chain reaction, DFS: disease free survival, HCC: hepatocellular carcinoma, HR: hazard ratio, LNA: Locked Nucleic Acid Clam, MSP: methylation specific PCR, n: number of patients, NGS: next-generation sequencing, OR: odds ratio, OS: overall survival, r: Pearson’s coefficient of correlation, RFLP: restriction fragment length polymorphism, RFS: recurrence free survival, seq: sequencing, SOMA: short oligonucleotide mass spectrometry analysis, tiDNA: tissue-related DNA

**Table 2:** Circulating Tumor DNA in Pancreatic Carcinoma

Reference	Origin	PC (*n*)	Technique	Circulating markers	ctDNA mutation (%)	tiDNA mutation (%)	Mutation in liquid and tumor tissue			Carcinoma vs. healthy		Biomarker
							Sensitivity (%)	Specificity (%)	Concordance ctDNA/tiDNA (%)	Sensitivity (%)	Specificity (%)	
Riviere et al. (2018)	Plasma	25	NGS	KRAS MYC EGFR	NA	NA	NA	NA	96 94 91	NA	NA	NA
Jiao et al. (2007)	Plasma	83	PCR and Seq MSP	KRAS mppENK, mp16	62.7	NA	NA	NA	NA	NA	NA	NA
Patel et al. (2019)	Blood	112	NGS	KRAS TP53	43.8 45.8	90.9 77.3	NA	NA	52 61	NA	NA	ctDNA: OS (HR = 4.35; *p* = 0.001)
Watanabe et al. (2019)	Plasma	39	ddPCR	KRAS	30.8	88.1	NA	NA	NA	NA	NA	OS (HR = 54.5; *p* < 0.001)
Takai et al. (2015)	Plasma	259	ddPCR	KRAS	32	NA	NA	NA	NA	NA	NA	NA
Sugimori et al. (2020)	Plasma	45	ddPCR	KRAS	51.1	95.7	NA	NA	NA	NA	NA	Worse PFS
Groot et al. (2019)	Preop plasma	59	ddPCR	KRAS	49	NA	90	88	NA	NA	NA	RFS (HR = 2.67; *p* = 0.011); OS (HR = 2.37; *p* = 0.048)
Tjensvoll et al. (2016)	Plasma	14	PNA-clamp PCR	KRAS Pre-CTX	71.4	NA	NA	NA	NA	NA	NA	RFS (HR = 1.29; *p* = 0.014); OS (HR = 1.43; *p* = 0.01)
Chen et al. (2017)	Plasma	189	NGS	KRAS	93.7	NA	NA	NA	NA	NA	NA	TTP (HR = 1.45; *p* = 0.002); OS (HR = 1.45; *p* = 0.001)
Kinugasa et al. (2015)	Serum	66	ddPCR, PCR-PHFA	KRAS	62.6	74.7	76.8	78.9	77.3	NA	NA	OS (HR = 3.24; *p* = 0.001)
Adamo et al. (2017)	Plasma	26	NGS	KRAS	27	78	NA	NA	NA	NA	NA	OS (HR = 2.89; *p* = 0.018)
Maire et al. (2002)	Serum	47	PCR	KRAS	47	NA	NA	NA	NA	47	87	NA
Dianxu et al. (2002)	Plasma	41	PCR–RFLP Seq	KRAS	70.7	91.7	75.8	100.0	77.8	NA	100	NA
Pratt et al. (2019)	Plasma	17	ddPCR	KRAS	86	NA	86	70	NA	NA	NA	NA
Earl et al. (2015)	Plasma	31	ddPCR	KRAS	25.8	58.3	42.9	60	50	NA	NA	OS (HR = 12.2; *p* < 0.001)
Uemura et al. (2004)	Plasma	28	Mismatch ligation assay	KRAS	32.1	92.9	34.6	100.0	39.2	NA	100	NA
Marchese et al. (2006)	Plasma	30	PCR and Seq	KRAS	0	70	NA	NA	NA	NA	NA	NA
Park et al. (2018)	Plasma	17	Targeted-NGS and ddPCR	KRAS	58.8	76.5	NA	NA	NA	NA	NA	NA
Del Re et al. (2017)	Plasma follow-up during palliative CTX	27	ddPCR	KRAS	70.4	NA	NA	NA	NA	NA	NA	PFS (2.5 vs. 7.5 months; *p* = 0.03); OS (6.5 vs. 11.5 months; *p* = 0.009)
Perets et al. (2018)	Plasma	17	NGS	KRAS	29.4	NA	NA	NA	NA	NA	NA	OS (*r* = -0.76; *p* = 0.03)
Kim et al. (2018)	Plasma	106	ddPCR	KRAS	80.5	96.1	78.4	33.3	NA	NA	NA	PFS (HR = 2.08; *p* = 0.009) OS (HR = 1.97; *p* = 0.034)
Lin et al. (2018)	Plasma	65	ddPCR	KRAS	80	100	NA	NA	100	NA	NA	OS (HR = 3.1; *p* < 0.001)
Chen et al. (2010)	Plasma	91	PCR	KRAS	33	NA	NA	NA	NA	NA	NA	ctDNA: OS (HR = 7.39; *p* < 0.001)
Cohen et al. (2017)	Plasma	221	NGS	KRAS KRAS and four proteins	30 NA	NA NA	NA NA	NA NA	100 NA	NA 64	NA 99.5	NA
Nakano et al. (2018)	Serum	45	PNA clamp PCR	KRAS	24.4 (pre-op) 44.4 (post-op)	83.3	NA	NA	NA	NA	NA	RFS (HR = 2.92; *p* = 0.027)
Kruger et al. (2018)	Plasma	54	BEAMing	KRAS	67	58	75	100	79	83	100	Early CTX response prediction
Hadano et al. (2016)	Preop plasma	105	ddPCR	KRAS	31	82	NA	NA	100	NA	NA	OS (HR = 3.2; *p* < 0.001)
Dabritz et al. (2009)	Plasma	56	PNA clamp PCR	KRAS	36	100	NA	NA	NA	NA	NA	NA
Wu et al. (2014)	Plasma	36	COLD-PCR Sanger sec	KRAS	72.2	NA	80.6	87.5	NA	NA	NA	NA
Semrad et al. (2015)	Plasma	27	PCR	KRAS	37	78	NA	NA	NA	NA	NA	DFS (1.8 vs. 4.6 months; *p* = 0.014) OS (3.0 vs. 10.5 months; *p* = 0.003)
Sausen et al. (2015)	Plasma	51	ddPCR	KRAS	43	88	NA	99.9	NA	NA	NA	NA
Ako et al. (2017)	Serum, plasma	40	ddPCR	KRAS	48	93	NA	NA	NA	NA	NA	Poor prognosis
Van Laethem et al. (2017)	Plasma	60	BEAMing	KRAS	65	NA	NA	NA	NA	NA	NA	PFS (HR = 0.32; *p* = 0.002) OS (HR = 0.27; *p* = 0.001)
Wei et al. (2019)	Plasma	38	NGS	KRAS T53	84 60	NA	NA	NA	NA	NA	NA	Correlation with tumor burden post CTX
Pietrasz et al. (2017)	Plasma	135	NGS and ddPCR	KRAS TP53 SMAD4	41.3 22.1 7.7	NA	NA	NA	NA	NA	NA	ctDNA: OS (HR = 1.99; *p* = 0.016)
Zill et al. (2015)	Plasma	17	NGS	KRAS	58.8	64.7	100	100	100	NA	NA	NA
				TP53	52.9	58.8	90	100	94			
				APC	11.8	11.8	100	100	100			
				SMAD4	5.9	11.8	100	100	94			
				FBXW7	11.8	5.9	50	100	100			
				KRAS, TP53, APC, SMAD, and FBXW7	82.3	88.2	92.3	100	97.7			
Pishvaian et al. (2017)	Blood	23	NGS	KRAS T53 SMAD4 CDKN2A	29 NA 0 0	87 NA 26.1 47.8	NA	NA	39 26.1 0 0	NA	NA	NA
Yu et al. (2017)	panc juice	115	NGS	TP53 and SMAD4	64.7	NA	NA	NA	NA	64.7	100	NA
Kanda et al. (2013)	panc juice	43	PCR and Sanger sec	TP53	67.4	NA	NA	NA	NA	67.4	100	NA
Cheng et al. (2017)	Plasma	188	ddPCR NGS	BRCA2 KDR EGFR ERBB2 KRAS	11.7 13.8 13.3 13.3 72.3	NA	NA	NA	NA	NA	NA	ERBB2: OS (HR = 1.61; *p* = 0.035) KRAS: OS (HR = 1.45; *p* = 0.019)
Berger et al. (2018)	Plasma	20	NGS and ddPCR	KRAS and TP53	80	81.8	NA	NA	NA	80	NA	PFS (*r* = – 0.86; *p* = 0.01)
Henriksen et al. (2016)	Plasma	95	MSP	10 genes	NA	NA	NA	NA	NA	76	83	UICC stages I-II vs. III–IV (AUC = 0.82)
Liggett et al. (2010)	Plasma	30	microarray- mediated methylation	17 marker panel	NA	NA	NA	NA	NA	91.2	90.8	NA
Park et al. (2012)	Plasma	106	MSP	mNPTX2	80	NA	NA	NA	NA	80	76	NA
Melnikov et al. (2009)	Plasma	30	multiplexed array- methylation	mCCND2, mPLAU mSOCS1, mVHL, mTHBS1	NA	NA	NA	NA	NA	76	59	NA
Melson et al. (2014)	Plasma	30	MSP	mVHL, mMYF3, mTMS, mGPC3, mSRBC	NA	NA	NA	NA	NA	81	67	NA
Eissa et al. (2019)	Plasma	39	MSP	mBNC1	65.1	NA	NA	NA	NA	64.1	93.7	NA
				mADAMTS1	87.2					87.2	95.8	
				mBNC1 and mADAMTS1	97.4					97.4	91.6	
Yi et al. (2013)	Serum	42	MOB and MSP	mBNC1	92	NA	NA	NA	NA	79	89	NA
				mADAMTS1	68					48	92	
				mBNC1 and mADAMTS1	NA					81	85	

AUC: area under the curve, BEAMing: beads, emulsion, amplification, magnetics PCR, COLD-PCR: co-amplification-at-lower denaturation-temperature PCR, ctDNA: circulating tumor DNA, CTX: chemotherapy, ddPCR: droplet digital polymerase chain reaction, DFS: disease free survival, LNA: Locked Nucleic Acid, MSP: methylation specific PCR, MOB: methylation on beads, n: number of patients, NGS: next-generation sequencing, OS: overall survival, PC: pancreatic cancer, PNA: Peptide Nucleic Acid, panc: pancreatic, PFS: progression free survival, PHFA: preferential homoduplex formation assay, preop: preoperative, r: Pearson’s coefficient of correlation, RFLP: restriction fragment length polymorphism, RFS: recurrence free survival, seq: sequencing, SOMA: short oligonucleotide mass spectrometry analysis; TTP: time to progression, tiDNA: tissue-related DNA, UICC: Union for International Cancer Control

## Cell-free and circulating tumor DNA

Cell-free (cf) DNA originates from normal cells exported by exosomes as well as from apoptotic and necrotic cells with highly fragmented, double-stranded DNA of approximately 150–180 base pair fragments in size being released into the bloodstream. In 1948, Mandel and Metais first reported the presence of cfDNA in human circulation followed by detection in urine, saliva, and other body fluids (Botezatu et al. 2000; Liao et al. 2000; Mandel and Metais 1948; Mao et al. 1994). A recent study showed that most of cfDNA derive from bone-marrow and liver in healthy individuals (Sun et al. 2015). Examples for clinical applications of cfDNA are the non-invasive prenatal testing for chromosomal aneuploidies by fetal cfDNA in the plasma of pregnant women or the monitoring of graft rejection following organ transplantation by donor-derived cfDNA in the plasma of the recipients (Chiu et al. 2008; Lo et al. 1997, 1998; Snyder et al. 2011). In 1977, Leon et al. demonstrated that cancer patients had a relative higher level of cfDNA than healthy controls with increased levels after radiation therapy (Leon et al. 1977). It was postulated that cancer patients have higher levels of cfDNA than healthy individuals (Shapiro et al. 1983). However, cfDNA levels have also been linked to outcomes in patients with a variety of other physiological and pathological conditions, including exercise, inflammation, circadian rhythm, exposure to smoking, sepsis, and trauma (Aucamp et al. 2018). In fact, cfDNA is composed of both coding and non-coding genomic DNA that can be used to examine mutations and polymorphisms, microsatellite instability, and epigenetic methylation (Bruhn et al. 2000; Downward 2003; Grutzmann et al. 2008; Jahr et al. 2001; Schwarzenbach et al. 2011). Epigenetic changes are considered as an early event in carcinogenesis and might, therefore, be a suitable early diagnostic tumor marker.

Since the extraction of plasma DNA with the same genetic changes as the primary tumor in 1989 and identification of mutated KRAS sequences in the plasma or serum from patients with PC in 1994, circulating tumor (ct) DNA is becoming a research hotspot with high potential as liquid biopsy marker in cancer medicine (Sorenson et al. 1994; Stroun et al. 1989). CtDNAs as a part of circulating cfDNA are mutant DNA fragments released to the circulation by tumor cells of different cancer entities. CtDNA is considered to be released from an increasing proportion of necrotic and apoptotic cells in primary tumors, secondary deposits and circulating tumor cells, corresponding to an increase in ctDNA. The half-lives of ctDNAs range from 15 min to a few hours which enables ctDNA analysis to be considered as a ‘real-time’ snapshot of disease burden with supposed clearance through nuclease activity, renal excretion, and uptake by the liver and spleen (Diehl et al. 2008; Minchin et al. 2001; Tamkovich et al. 2006; Yu et al. 2013). CtDNA can be detected by tumor-specific mutants and are less impacted by intratumor heterogeneity than a single specimen of tumor tissue (Bettegowda et al. 2014; Diaz and Bardelli 2014; Fleischhacker and Schmidt 2007; Melo et al. 2015; Sausen et al. 2014). It is supposed, that ctDNA harbor the same (epi-) genetic alterations as the originating primary tumor cells. Recently, Heitzer et al. demonstrated the impact of ctDNA in early detection, surveillance, and personalized treatment in different cancer entities like colorectal, breast, and non-small cell lung cancer (Heitzer et al. 2015). In addition, previous studies have indicated a positive correlation between ctDNA levels and tumor burden in various cancer types with increasing copy numbers of ctDNA per mL plasma in advanced and metastatic tumors, as well as different methylation status of ctDNA to normal cfDNA and blood leukocytes (Bettegowda et al. 2014; Guo et al. 2017; Madic et al. 2012; Xu et al. 2017). Furthermore, it is hypothesized that ctDNA could be a signaling trigger for cancer progression by horizontal DNA transfer affecting the biology of host cells (Bergsmedh et al. 2001; Gahan and Stroun 2010; Garcia-Olmo et al. 1999). Thus, ctDNA levels may serve as an early biomarker for diagnostic and therapy monitoring, prior to clinically or radiographically measureable changes of tumor burden in patients. However, ctDNAs represent a variable fraction of cfDNAs ranging from 0.01% to more than 50% in cancer patients (Diaz and Bardelli 2014; Diehl et al. 2008). Deoxyribonuclease activity and the release of cfDNA by normal cells in peripheral circulation reduces ctDNA concentrations. Therefore, the detection of ctDNA is challenging and necessitates the standardization of extraction procedures to be able to distinguish ctDNA from the large amount of cfDNA.

## Next-generation sequencing and digital droplet PCR

In the last 2 decades, growing knowledge on non-coding genome functionality and genome-wide sequence variation has improved personalized medicine and molecular oncology. With technological advances broadly categorized into PCR-based and genomic sequencing-based techniques, sensitivity and specificity of biomolecular techniques for ctDNA detection have been highly improved. Currently, high-throughput next-generation sequencing (NGS) and digital droplet PCR (ddPCR) are the most promising methods for the detection of mutations in liquid biopsies. In general, plasma samples are used in preference over serum because of lower concentrations of wild-type DNA (Jung et al. 2003). NGS techniques allow the simultaneous assessment and detection of multiple genetic aberrations and copy number changes, including targeted techniques such as enhanced tagged amplicon deep sequencing, or whole-exome sequencing. The aim of NGS is to generate extensive information about the mutation landscape, then to screen the genome and discover new genomic aberrations, e.g. those that confer resistance to a specific targeted therapy (Murtaza et al. 2013). The whole-genome and whole-exome sequencing could provide more comprehensive information regarding the mutant status of ctDNA. However, the targeted region deep sequencing, which focuses on a fewer gene loci with ultra-deep sequencing, has gained popularity, because the sequencing region can be customized according to cancer types, sequencing purpose, costs, and turnaround time (Leary et al. 2010; Martinez et al. 2013). Among PCR-based techniques, digital PCR including droplet PCR and BEAMing (beads, emulsion, amplification, magnetics) PCR appears as the most promising approach for detection of highly recurrent hotspot mutations at frequencies as low as 0.01%. Compared to quantitative real-time (qRT) PCR, samples are processed with a water–oil emulsion to allow for individual droplets to be assessed as a discrete PCR sample and does not rely on external calibrant. DNA templates are distributed into thousands of droplets each containing only one DNA fragment. Digital PCR has a higher tolerance for enzyme-inhibiting substances thereby improving sensitivity and specificity of mutant DNA detection (Hindson et al. 2011; Zhang et al. 2015). In combination with circulating nucleic acids, ddPCR has gained wide applicability in liquid biopsy diagnostic of cancer, especially in the assessment of methylation status to identify epigenetic dysregulation during carcinogenesis and measurement of changes in gene expression to early diagnosis of cancer, the absolute quantification of copy number variations to predict disease progression, or the detection of rare mutations within ctDNA to guide targeted therapy (Huggett et al. 2015).

## Genomic alterations of ctDNA in HCC

NGS profiling of surgically resected HCC revealed a highly heterogeneous cancer caused by the accumulation of genomic and epigenomic alterations (Ozen et al. 2013). Recently, integrative genomic characterization by whole exome sequencing and analyses of DNA copy number, DNA methylation, RNA, microRNA, and proteomic expression defined two major molecular subtypes of HCC. One subtype is defined as a proliferation class associated with higher HBV prevalence and poor clinical outcome characterized by activation of proliferative signaling pathways such as Phosphatidylinositide-3-kinase/AKT/mammalian target of rapamycin and RAS- Mitogen-Activated Protein Kinase pathways, Wingless-type (WNT)/Transforming Growth Factor (TGF) beta signaling, amplification of mitogenic Fibroblast Growth Factor family members and of the cell cycle regulatory subunit Cyclin D1 (CCND1) and inactivation of the tumor suppressor TP53. The second subtype contains a heterogenous non-proliferation class associated with higher HCV prevalence or alcohol abuse and a better clinical outcome characterized by WNT–beta–catenin pathway activation via Catenin Beta 1 (CTNNB1) mutation, Telomerase Reverse Transcriptase (TERT) promotor mutation and silencing of the tumor suppressor Cyclin Dependent Kinase Inhibitor 2A (CDKN2A) by mutation and DNA methylation (Comprehensive and Integrative Genomic Characterization of Hepatocellular Carcinoma 2017). This comprehensive and integrative characterization of molecular profiling in HCC tissues followed by the identification and quantification of corresponding ctDNA in the plasma may provide powerful data for targeted therapies and monitoring of therapy response.

Several genomic and epigenomic alterations within ctDNA as molecular targets in the context of aberrant signaling pathways were detected by NGS, ddPCR and methylation-specific PCR (MSP) in HCC patients (Table 1). TERT promotor mutations were found to be the most common point mutations in several carcinoma entities with reactivation of telomerase enabling limitless cell proliferation driven by oncogenes (Bell et al. 2016). In HCC, TERT promotor mutations were found in dysplastic nodules and early stages with a reported frequency of 59–90% correlating with poor survival (Nault and Villanueva 2015). DdPCR by Huang et al. and NGS by Liao et al. of TERT mutation (c.1-124C > T) in plasma ctDNA of HCC patients revealed a frequency of 23% and 5% with a high specificity of 85% and 95%, respectively (Huang et al. 2016; Liao et al. 2016). Aberrant amplification of TERT was reported to be significantly associated with CTNNB1 mutations in HCC, indicating that the interaction between upregulation of TERT mutations and dysregulation of the WNT–beta–catenin pathway could promote hepatocellular carcinogenesis (Nault et al. 2013). Gain of function mutations in the CTNNB1 gene encoding beta catenin allow the accumulation of beta catenin within the cell nucleus through WNT pathway und promote tumor progression in about 30% of cases in HCC. CtDNA analysis on CTNNB1 mutations (c.121A > G, c.122C > T, c.133T > C, c134C > T) resulted in a high specific frequency of about 10% in two small HCC collectives screened by ddPCR and NGS (Huang et al. 2016; Liao et al. 2016). Another well known driver gene of HCC is TP53 with a high mutation frequency of more than 30%. Mainly missense mutations in the DNA-binding domain of TP53 are generally thought to abrogate the tumor suppressor function of p53 as the guardian of the genome. Loss of p53 function with consecutive dysregulation of apoptosis, cell cycle arrest, DNA repair and metabolic regulation is a prerequisite for tumor initiation and progression in a multitude of human cancers. However, mutant p53 not only lose tumor suppressive functions of wild-type p53 but also gain new oncogenic properties promoting tumor cell proliferation, angiogenesis, and metastasis. The mechanism for the accumulation of mutant p53 and its mutational gain of function in tumors is not yet well understood. The frequency of mutant TP53 in blood and urine ctDNA of HCC studies ranged between 5 and 60% (An et al. 2019; He et al. 2019; Huang et al. 2016; Ikeda et al. 2018a, b; Kaseb et al. 2019; Kimbi et al. 2005; Liao et al. 2016; Lin et al. 2011; Marchio et al. 2018; Riviere et al. 2018; Szymanska et al. 2004; Xiong et al. 2019). Liao et al. could reveal that the median recurrence-free survival (RFS) of patients with the presence of TERT, CTNNB1 and TP53 mutations detected in ctDNA post surgical treatment was significantly decreased with 3 versus 12 months (Liao et al. 2016). Similarly, postoperative detection of ctDNA related mutations by An et al. with TP53 as the most common mutant gene correlated significantly with worse disease-free survival (DFS) of 6.7 versus 17.5 months (An et al. 2019).

## Methylation alterations of ctDNA in HCC

Besides genetic alterations with change of DNA sequence, epigenetic silencing of tumor suppressor genes by promotor hypermethylation has been proven to be present in precursor lesions of HCC. Aberrant DNA hypermethylation consists of the addition of a methyl residue on cytosines preceding guanosines leading to a condensed chromatin structure without transcriptional activity. High concordance of DNA methylation in plasma and tumor DNA could be shown for a number of tumor suppressor genes in HCC (Iyer et al. 2010). Aberrant methylation of the cyclin-dependent kinase inhibitor genes p15 (CDKN2B or p15INK4b) and p16 (CDKN2A or p16INK4a) on chromosome 9p21 in the peripheral circulation of HCC patients using MSP is one of first detected epigenetic changes associated with hepatocellular tumorigenesis. Wong et al*.* detected concurrent p15 and p16 methylation in 74% of ctDNA of 23 blood samples from 92% of HCC patients with tumor p15/p16 methylation (Wong et al. 1999, 2000). High incidence of p16INK4a promoter hypermethylation in ctDNA with significant decrease in postoperative blood samples was shown to be a useful marker in the detection and monitoring of HCC (Wong et al. 2003). Correspondingly, Huang et al. demonstrated higher levels of methylated p16INK4a in circulating cfDNA of 66 HCC serum samples versus 43 benign chronic liver diseases (Huang et al. 2014). Further common tumor suppressing and cell cycle regulation-related genes with promoter hypermethylation are the adenomatous polyposis coli (APC) on chromosome 5q21 and the Ras association domain family protein 1A (RASSF1A) genes on chromosome 3p21.3 with high frequency of promoter hypermethylation in tumor and blood samples of HCC patients (Hu et al. 2010; Huang et al. 2011; Yeo et al. 2005). Mohamed et al. as well as Mansour et al. could demonstrate that serum levels of methylated RASSF1A could well discriminate HCC patients from healthy volunteers and from chronic HCV infection with an incidence of about 90% in HCC serum samples (Mansour et al. 2017; Mohamed et al. 2012). Similar frequencies of methylated RASSF1A in serum of HCC patients at diagnosis or 1 year after tumor resection versus low concentrations in HBV carriers correlated with poorer disease-free survival (DFS) in a study of Chan et al. (Chan et al. 2008). Moreover, elevated plasma methylation levels of APC or RASSF1A correlated with poorer overall survival reaching significance for RASSF1A in multivariate analysis (Huang et al. 2011). Coevaluation of RASSF1A and Long Interspersed Nucleotide Element 1 (LINE-1) as one of the major repetitive DNA sequence of the human genome and most active mediator of retrotransposition revealed LINE-1 hypomethylation in 66.7% and RASSF1A promoter hypermethylation in 73.3% only in HCC serum DNA samples correlating with early recurrence and poor survival after curative resection (Liu et al. 2017). Tangkijvanich et al. described significantly increased serum LINE-1 hypomethylation in HCC as independent prognostic factor of overall survival (Tangkijvanich et al. 2007). Combined detection of ctDNA methylation markers was performed by several studies to improve the efficiency in early HCC diagnostic. Plasma methylation analysis of the four genes panel with APC, glutathione *S*-transferase P 1 (GSTP1), RASSF1A, and secreted frizzled-related protein 1 (SFRP1) resulted in an increased accuracy of 93% to differentiate between HCC and healthy controls (Huang et al. 2011). Xu et al. constructed a diagnostic prediction model using a cfDNA methylation marker panel that predicted HCC survival and could effectively discriminate patients with HCC from individuals with HBV/HCV infection, fatty liver disease as well as healthy controls superior to AFP (Xu et al. 2017). Although a multitude of aberrant methylated genes could be identified as prognostic target in HCC, there is no recognized biomarker confirmed in multiple centers (Han et al. 2014; Iizuka et al. 2011; Ji et al. 2014; Oussalah et al. 2018; Sun et al. 2013; Wang et al. 2006; Wen et al. 2015; Wu et al. 2017; Zhang et al. 2013).

## Genomic alterations of ctDNA in PC

In the last decade, comprehensive genomic analysis allowed important advances in the understanding of the molecular pathogenesis of PC with reclassification in different specific subtypes (Bailey et al. 2016; Biankin et al. 2012; Collisson et al. 2011; Jones et al. 2008; Moffitt et al. 2015; Waddell et al. 2015; Witkiewicz et al. 2015). Several studies using different techniques could reveal that reproducible molecular subgroups with consistent alterations in genes and signaling pathways are emerging in PC. Evaluating 456 specimens of resected PC by a combination of whole-genome sequencing and deep-exome sequencing, Bailey et al. identified genetic mutations particularly of KRAS in 92%, cell cycle checkpoint mutations as TP53 and CDKN2A in 78%, aberrations in TGF beta signaling as SMAD4, TGFBR1, and Activin A receptor 1B in 47%, mutations leading to histone modification in 24%, mutations in the Breast Cancer Gene (BRCA) pathway in 17%, and mutations in the ATP-dependent chromatin remodeling complex as AT-rich interaction domain 1A (ARID1A) in 14% (Bailey et al. 2016). However, there is still a lack of consensus in the clinical applicability of current PC subtyping approaches. Recently, plasma cfDNA profiling in 38 patients with advanced PC receiving first-line FOLFIRINOX chemotherapy could demonstrate that 65.8% of patients had at least one common driver gene alteration in KRAS, TP53, SMAD4, or CDKN2A in high concordance with corresponding tumor tissue (Table 2) (Wei et al. 2019). Interestingly, the dynamics of total cfDNA concentration correlated positively with tumor burden following chemotherapy and might be a promising tool for early response prediction and therapy surveillance in patients with advanced PC. Among these key genes, KRAS is the best characterized tumor-related gene in PC with the highest frequency of KRAS point mutations located in codon 12 and with appearance even at early stages of PC carcinogenesis (Almoguera et al. 1988; Rhim et al. 2014; Uemura et al. 2003). Therefore, KRAS mutant ctDNA represents a promising biomarker and therapeutic target of PC. Using ddPCR and targeted NGS, different KRAS mutations were detected in up to 80% of PC serum and plasma samples and were associated with decreased survival (Adamo et al. 2017; Ako et al. 2017; Chen et al. 2010; Cohen et al. 2017; Dabritz et al. 2009; Del Re et al. 2017; Dianxu et al. 2002; Earl et al. 2015; Hadano et al. 2016; Jiao et al. 2007; Kim et al. 2018; Kinugasa et al. 2015; Kruger et al. 2018; Lin et al. 2018; Maire et al. 2002; Marchese et al. 2006; Nakano et al. 2018; Park et al. 2018; Patel et al. 2019; Perets et al. 2018; Pratt et al. 2019; Riviere et al. 2018; Sausen et al. 2015; Semrad et al. 2015; Sugimori et al. 2020; Takai et al. 2015; Tjensvoll et al. 2016; Uemura et al. 2004; Van Laethem et al. 2017; Watanabe et al. 2019; Wu et al. 2014). Chen et al. published a KRAS mutant ctDNA detection rate of 93.7% which correlates with time to progression and overall survival of 189 patients with unresectable PC (Chen et al. 2017). In metastatic PC absence of KRAS mutant ctDNA was significantly associated with survival benefit of 37.5 versus 8 months (*p* < 0.004) (Perets et al. 2018). Correspondingly, PC patients with KRAS mutant ctDNA were more likely to relapse after curative surgery than those without KRAS mutant ctDNA with desease-free survival of 6.1 versus 16.1 months and overall survival of 13.6 versus 27.6 months (*p* < 0.001) (Groot et al. 2019; Hadano et al. 2016; Sausen et al. 2015). Serial plasma testing of KRAS mutant ctDNA in advanced PC patients receiving chemotherapy allowed the monitoring of rapid changes of KRAS mutant ctDNA levels superior to CA19-9 and CEA kinetics (Kruger et al. 2018). Targeting KRAS pathway is a promising effort to make therapeutic progress in PC (Krantz and O’Reilly 2018). Although mutant KRAS is often identified in plasma as a ctDNA benchmark for PC, advances in sequencing of the whole PC genomic landscape have expanded the panel of key mutations (Kanda et al. 2013; Pietrasz et al. 2017; Pishvaian et al. 2017; Yu et al. 2017; Zill et al. 2015). CtDNA whole-exome sequencing of 60 hotspot genes in metastatic PC identified KRAS, BRCA2, KDR, EGFR, and ERBB2 as candidate genetic mutations. DdPCR could validate ctDNA mutations at ERBB2 exon17 and KRAS G12V that were significantly correlated with worse overall survival (Cheng et al. 2017). Berger et al. performed NGS and ddPCR to dynamically monitor the most frequently ctDNA mutated genes in PC. TP53 and KRAS mutation levels were significantly decreased during treatment, and on the other hand significantly increased during tumor progression correlating with progression-free survival (Berger et al. 2018). However, current biomolecular technologies confirm the genetic heterogeneity of PC with a large number of low-frequently mutated loci and a large fraction of patients who does not harbor mutations in KRAS or TP53 (Martinez et al. 2013).

## Methylation alterations of ctDNA in PC

Methylation analyses of ctDNA that reveal epigenetic alterations with more or less diagnostic and prognostic impact reflect the remarkable heterogeneity in PC patients. CfDNA promotor hypermethylation in plasma or serum could be detected in all stages of PC (Henriksen et al. 2017b). Henriksen et al. developed a survival prediction model based on plasma-derived cfDNA hypermethylation of a large gene panel that enables the stratification of patients into risk groups (Henriksen et al. 2017a). Further methylation analyses of ctDNA were able to differentiate PC from chronic pancreatitis and healthy controls (Henriksen et al. 2016; Liggett et al. 2010; Melnikov et al. 2009; Melson et al. 2014; Park et al. 2012). Confirming study results of Yi et al., Eissa et al. could show that a two-gene promotor methylation panel of Zinc finger protein basonuclin-1 (BNC1) and A disintegrin and metalloproteinase with thrombospondin motifs 1 (ADAMTS1) increased sensitivity to 97.4% and specificity to 91.6% in plasma cfDNA of early PC stages (Eissa et al. 2019; Yi et al. 2013). So far, in relatively, few PC patients no single ctDNA promotor hypermethylation with adequate sensitivity and specificity has been found. Furthermore, serial ctDNA studies following the methylation profile of PC patients in accordance with treatment and tumor recurrence are lacking.

## Challenges in clinical utility of circulating DNA

CtDNA has gained considerable attention as novel liquid biopsy marker for cancer detection in asymptomatic individuals and of residual disease. Indeed, ctDNA has huge clinical potential for prognostication and response monitoring of patients with HCC and PC characterized by high tumor heterogeneity and dismal prognosis. However, although literature regarding ctDNA assays and molecular profiling is rapidly growing, its translation into clinical applicability is highly complex. Limited data are available regarding the blood draw procedure and pre-analytical variables that increase degradation of cfDNA or contamination by cellular genomic DNA derived from leukocyte lysis (Lee et al. 2001). Indeed, varying cfDNA purification methods and various protocol modifications may affect cfDNA yield and purity. There is consensus that cfDNA analysis requires special processing and handling by using cell-stabilization tubes and avoiding repeated freeze–thaw cycles. Furthermore, patient-related factors as medical treatment, smoking, exercise, age-related clonal hematopoiesis, inflammation or cardio-pulmonary disorders may contribute to the release of cfDNA. Interestingly, several studies could demonstrate that false-positive plasma genotyping is due to clonal hematopoiesis with non-malignant mutations harbored by hematopoietic cells with increasing frequency in 10% of patients over the age of 65 years, but only in 1% of patients under the age of 50 years (Hu et al. 2018; Jaiswal et al. 2014). The proportion of ctDNA as a fraction of cfDNA varies substantially between different patients, and different subclonal variants might be identified. Therefore, allele fractions of variants in ctDNA need to be interpreted with great caution.

In the last decade, numerous platforms for genotyping of cfDNA have been developed. However, the test characteristics of each platform as ddPCR and NGS vary and were validated in different patient populations with different lower limits of detection. Therefore, direct comparison of these platforms with reported high diagnostic specificity, but modest sensitivity is challenging and requires rigorous cross-assay comparisons. The low diagnostic sensitivity of ctDNA tests in carcinomas could be a major reason of discordant tissue and ctDNA genotyping results. Although clinical utility of ctDNA assays is mainly based on retrospective analyses, first FDA-approved application in 2018 for cfDNA assay in routine clinical practice could demonstrate high concordance between plasma and tumor tissue genotyping for early detection of specific EGFR mutation (T790M) and therapy stratification in advanced non-small cell lung cancer patients (Zhang et al. 2018). The concept of plasma genotyping is highly promising, although its application in the clinical routine to identify and treat patients with HCC or PC requires ongoing evaluation.

## Conclusion

Overall, ctDNA mapping of somatic driver mutations and specific epigenetic alterations has great potential in early detection and dynamic monitoring of hepatic or pancreatic carcinomas to achieve a significant decrease of mortality. However, low sensitivity of current ctDNA assays rest a major challenge.

So far, ctDNA analysis in PC and HCC could reveal high frequency of common key mutations as TP53 and CDKN2A. CTNNB1 and TERT mutations and aberrant methylation of RASSF1A and CDKN2A were detected in ctDNA of HCC patients, whereas high frequency of KRAS mutations was characteristic for PC. Prospective trial data based on sufficient sample size and defined entry criteria regarding the blood draw procedure and pre-analytical variables as well as standardization of experimental techniques that demonstrate the clinical utility of ctDNA assays in PC and HCC are required.

## Data Availability

All data generated or analyzed during this study are included in this published article.

## Abbreviations

ADAMTS1: A disintegrin and metalloproteinase with thrombospondin motifs 1
AFP: Alpha-fetoprotein
APC: Adenomatous polyposis coli
ARID1A: AT-rich interaction domain 1A
BNC1: Basonuclin-1
BRCA: Breast cancer gene
CA: Carbohydrate antigen
CCND1: Cyclin D1
CDKN2A: Cyclin-dependent kinase inhibitor 2A
CEA: Carcinoembryonic antigen
Cf: Cell-free
Ct: Circulating tumor
CTNNB1: Catenin Beta 1
ddPCR: Digital droplet PCR
DFS: Disease-free survival
EGFR: Epidermal growth factor receptor
5-FU: 5-Fluorouracil
GSTP1: Glutathione *S*-transferase P 1
HBV: Hepatitis B virus
HCC: Hepatocellular carcinoma
HCV: Hepatitis C virus
KRAS: Kirsten rat sarcoma viral oncogene homolog
LINE-1: Long interspersed nucleotide element 1
MSP: Methylation-specific PCR
NGS: Next-generation sequencing
PC: Pancreatic cancer
PDGFR: Platelet-derived growth factor receptor
qRT: Quantitative real time
RASSF1A: Ras association domain family protein 1A
RFS: Recurrence-free survival
SFRP1: Secreted frizzled-related protein 1
TERT: Telomerase reverse transcriptase
TGF: Transforming growth factor
VEGFR: Vascular endothelial growth factor receptor
WNT: Wingless-type
